# Supplementary material for: PIWIL3 Forms a Complex with TDRKH in Mammalian Oocytes
Source: Cells. 2020 May 29;9(6):1356. doi: 10.3390/cells9061356 (PMC7349845; doi:10.3390/cells9061356)
Supplement: Supplementary file 1 [file cells-09-01356-s001.zip › Supplementary material Tan et al.pdf]

Supplementary Information

PIWIL3 forms a complex with TDRKH in mammalian oocytes.

Minjie Tan[1], Helena T.A. van Tol[1], David Rosenkranz[2], Elke F. Roovers[3], Mirjam J. Damen[4,5], Tom A.E. Stout[1,6], Wei Wu[4,5,*], Bernard A.J. Roelen[1,6,*]

[1]Department of Farm Animal Health, Faculty of Veterinary Medicine, Utrecht University, Utrecht, the Netherlands.

[2]Johannes Gutenberg-University Mainz, Institute of Organismic and Molecular Evolution, Anselm-Franz-von-Bentzel-Weg 7, 55128 Mainz, Germany.

[3]Biology of Non-coding RNA Group, Institute of Molecular Biology (IMB), Ackermannweg 4, 55128 Mainz, Germany.

[4]Biomolecular Mass Spectrometry and Proteomics, Bijvoet Center for Biomolecular Research and Utrecht Institute for Pharmaceutical Sciences, Utrecht University, Utrecht, the Netherlands.

[5]Netherlands Proteomics Centre, Utrecht, the Netherlands.

[6]Department of Equine Sciences, Faculty of Veterinary Medicine, Utrecht University, Utrecht, the Netherlands.

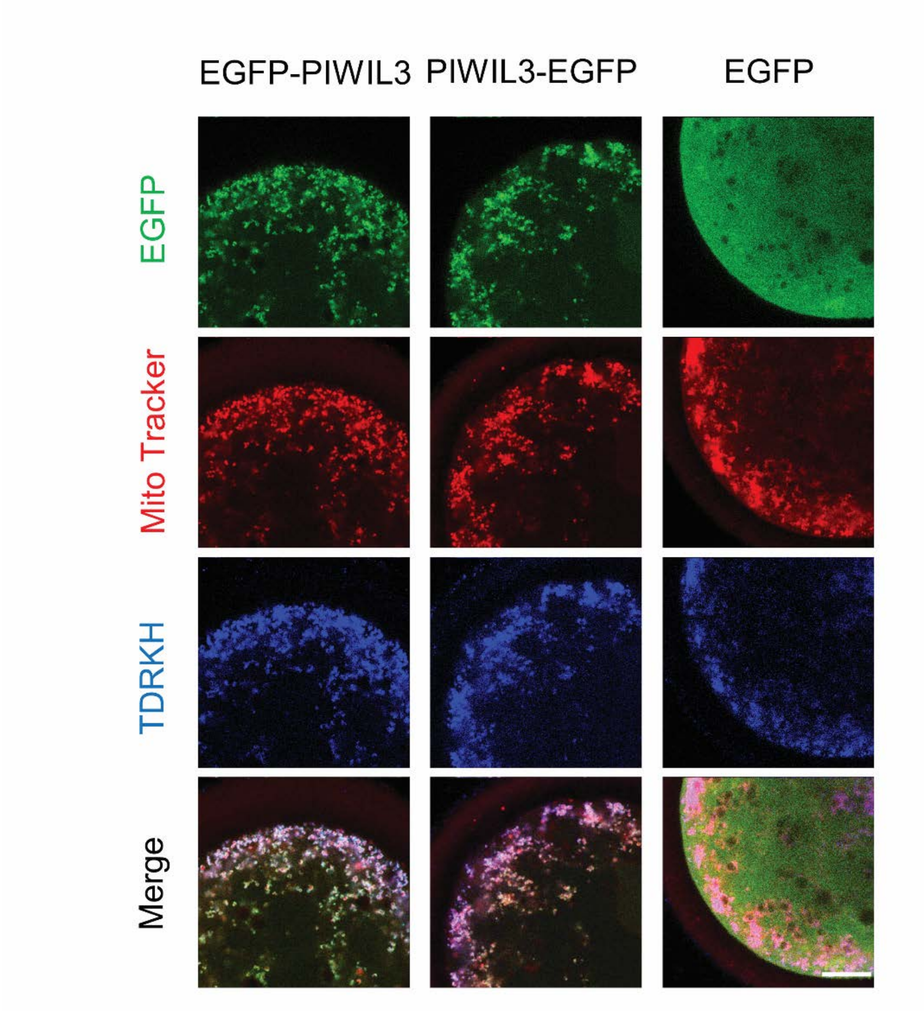

Fig. S1: Fluorescent localization after microinjection of PIWIL3-EGFP or EGFP-PIWIL3 mRNA into GV stage bovine oocytes. Distribution of EGFP-PIWIL3 and PIWIL3-EGFP (green), MitoTracker (mitochondria, red) and TDRKH (blue) in oocytes. Scale bar, 20 µm.

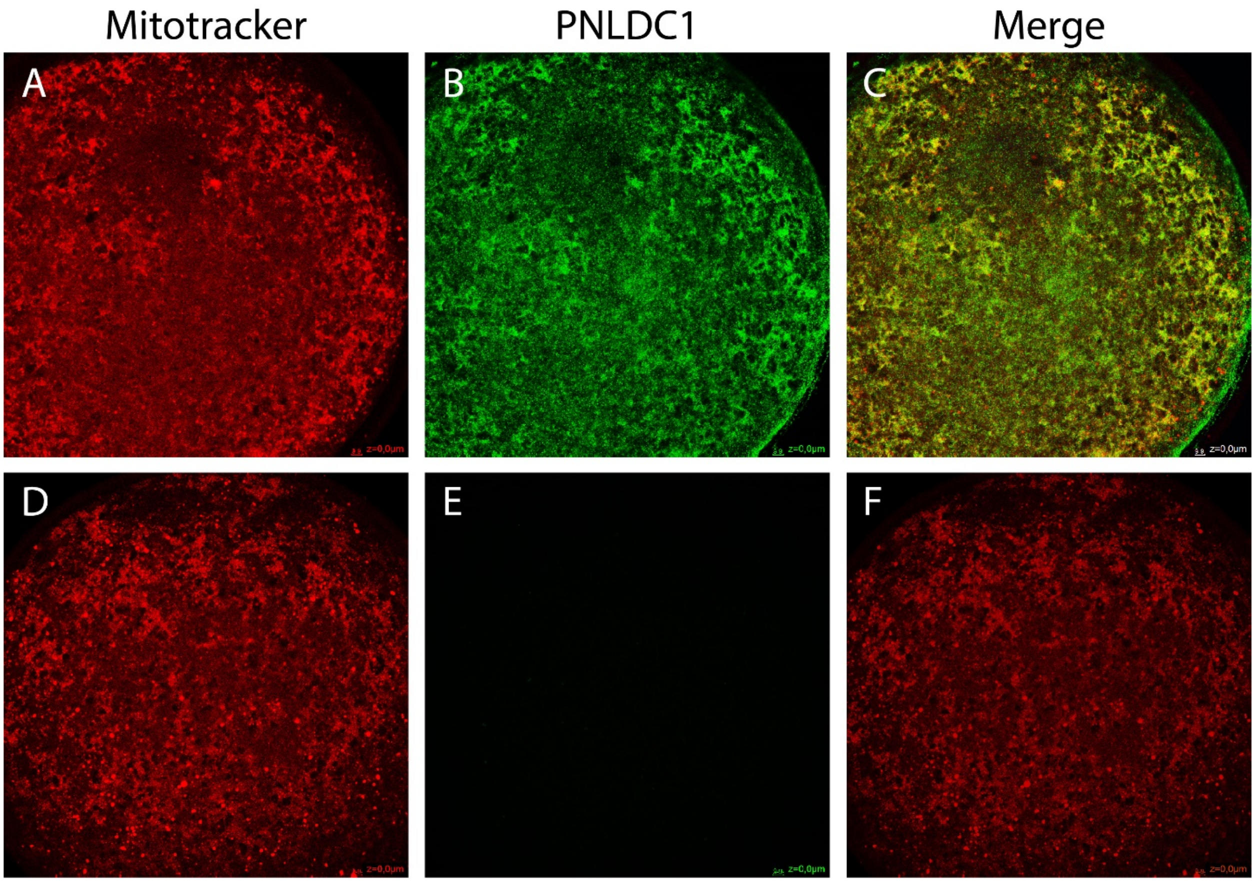

Figure S2: PNLDC1 colocalizes with mitochondria in oocytes. Distribution of mitochondria (A,D, Mitotracker, red), PNLDC1 (B green) and merge (C, F) in germinal vesicle stage oocytes. Panel E shows the same oocyte as in D, E, stained with PNLDC1 antibody but without secondary antibody. Scale bar, 20 µm.

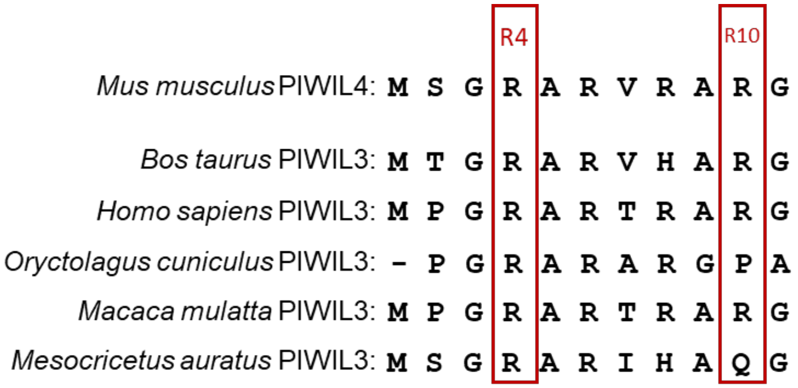

Figure S3: Sequence alignment of mammalian PIWI N terminal domain. R4 and R10 illustrate the arginines in *Bos taurus* PIWIL3 mutated in our experiment.

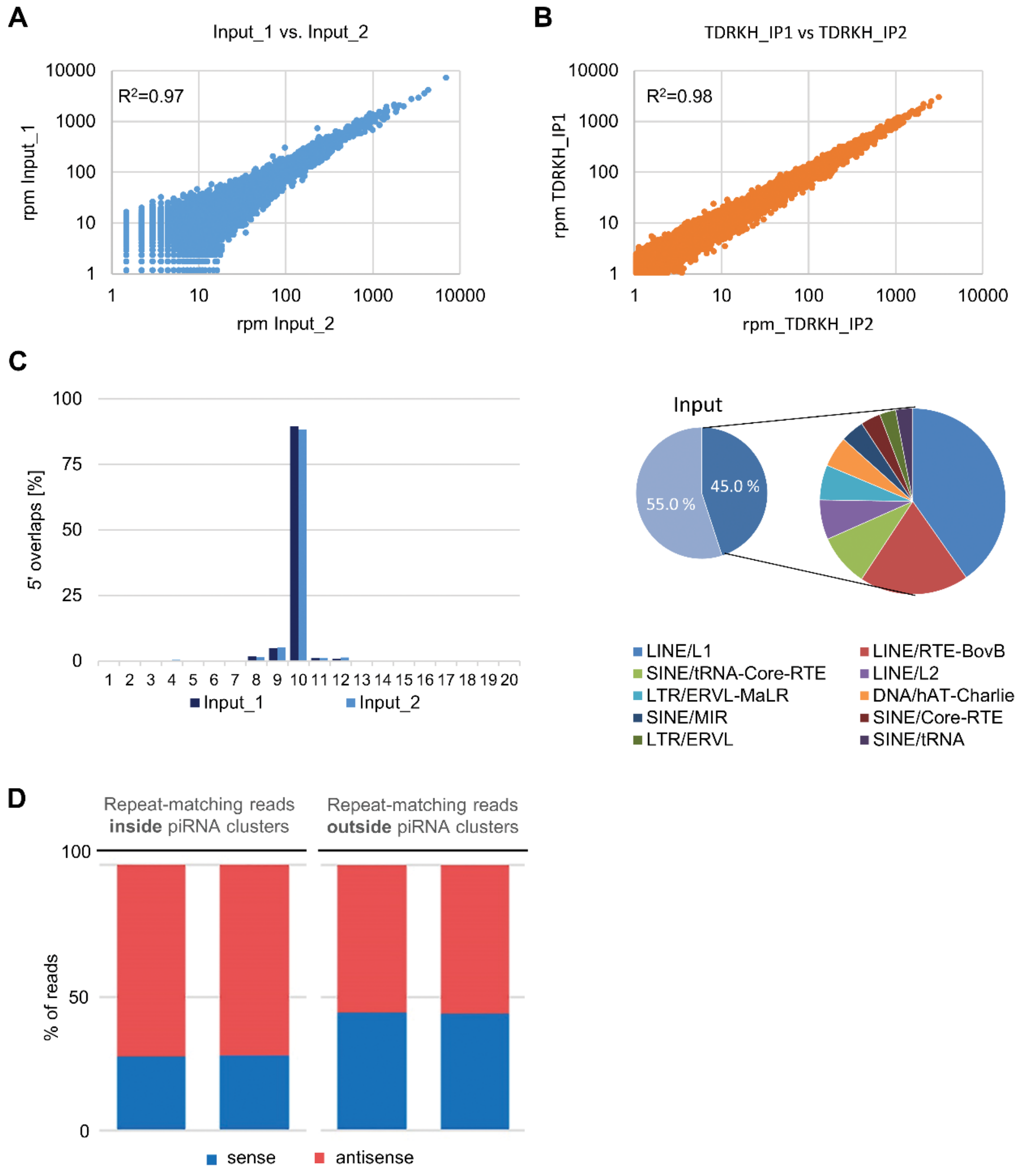

Figure S4: piRNA sequencing summary statistics. A) Technical reproducibility. Pearson correlation between sequencing replicates varied in the range of $0.97<r^2<0.98$. rpm, reads per

million. B) Overlaps of 5’ ends of reads that are mapped to opposite strands of the same locus.

C) Pie chart depicting the transposon content of bovine piRNA populations from Input samples.

D) Repeat-matching reads inside and outside piRNA clusters for Input samples.

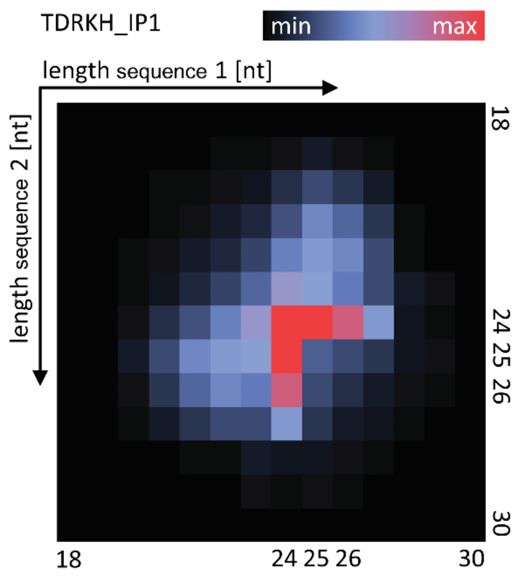

Figure S5: Sequence length analysis of piRNAs that participate in the ping-pong amplification loop. Ping-pong matrices illustrate frequent length-combinations of ping-pong pairs (sequences with 10 bp 5' overlap), indicated in red. X-axis and Y-axis refer to sequence read length of the two sequences of a ping-pong pair.

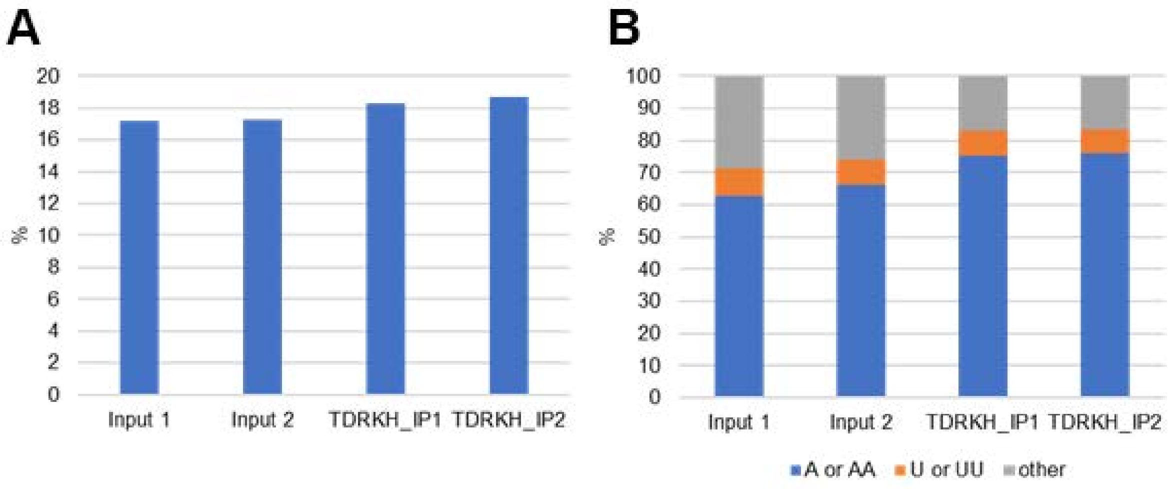

Figure S6: Non-templated nucleotide analysis. A) Frequencies of non-templated nucleotides at the 3' end from the indicated libraries. B) Frequencies of the identified non-templated nucleotides into "A", "U" or "other" tails.

Table S2: Primers for PIWIL3 (a) plasmid construction and (b) qRT-PCR. "f" and "r" indicate forward and reverse respectively.

a

Gene	Accession number	Sequence (5'-3')	Direction	Annealing temperature (°C)
PIWIL3	XM_010814123.1	CGGGGTACCAGAGGAGGAAAGACGGAAG<br>GGCGATATCCCCATTGTGCTTTCTTCTG	f<br>r	66
PIWIL3 De1	XM_010814123.1	AGACGGCGGGATACACCA<br>AGTCATTGCTGTCCTGAATCCG	f<br>r	68
PIWIL3 Mu1	XM_010814123.1	TCACGCCAAAGGCAGACGGCGGGATACA<br>ACTCTGGCCTTGCCAGTCATTGCTGTCCTG	f<br>r	67
PIWIL3 Mu2	XM_010814123.1	CAGCCTAAACTCCAGTCATTGACAGGTGTTAG<br>GATTTGTTTAGGTTGCTGAGCCGCTGT	f<br>r	66

b

Gene	Accession number	Sequence (5'-3')	Direction	Annealing temperature (°C)
PIWIL3	XM_010814123.1	AGAAGGAGCTTCGAGACTGG<br>GATTCTGCTGCAAGGTCAGG	f<br>r	61
TDRKH	NM_001105375.1	GCAAAGCGCGCGAAGGCTAAC<br>CCCACGGATCTCTGGGGGAC	f<br>r	58
PNLDC1	XM_015464886.1	TGCAAGGGGCTTTTCTGTGT<br>CCAGAGTTCTTGGTGGGGTT	f<br>r	60
GAPDH	NM_001034034.2	AGGCCATCACCATCTTCCAG<br>GGCGTGGACAGTGGTCATAA	f<br>r	61
SDHA	NM_174178	GCAGAACCTGATGCTTTGTG<br>CGTAGGAGAGCGTGTGCTT	f<br>r	64

Table S1 TDRKH IP MS data (Excel file).

Table S3: Small RNA sequencing data (Excel file).

Table S4: Non-transposon putative target transcript genes (Excel file).
