# Supplementary figures and images for: PIWIL3 Forms a Complex with TDRKH in Mammalian Oocytes
Source: Cells. 2020 May 29;9(6):1356. doi: 10.3390/cells9061356 (PMC7349845; doi:10.3390/cells9061356)

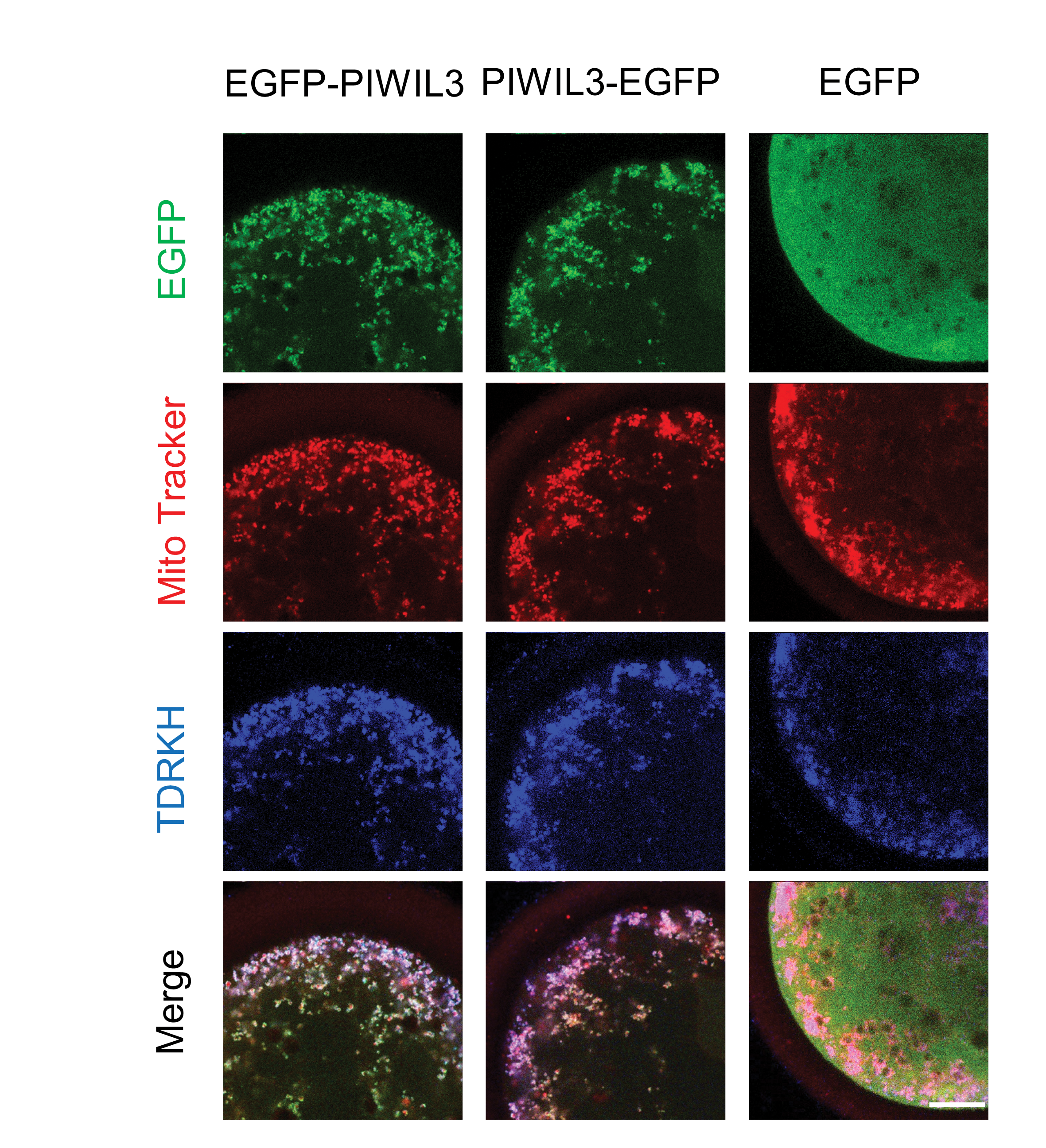

Supplement: Supplementary file 1 [file cells-09-01356-s001.zip › fig. S1.tif]

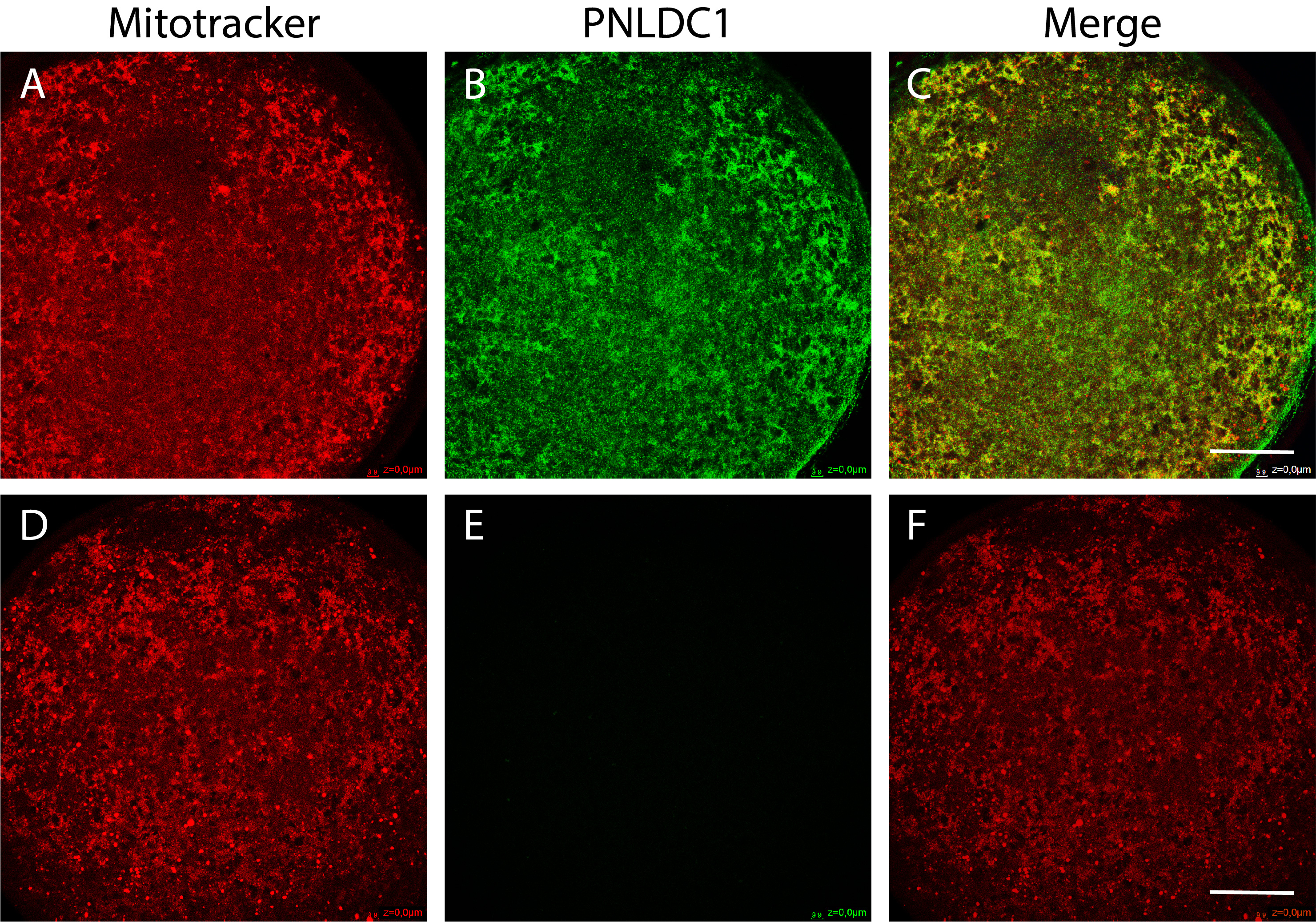

Supplement: Supplementary file 1 [file cells-09-01356-s001.zip › fig. S2.tif]

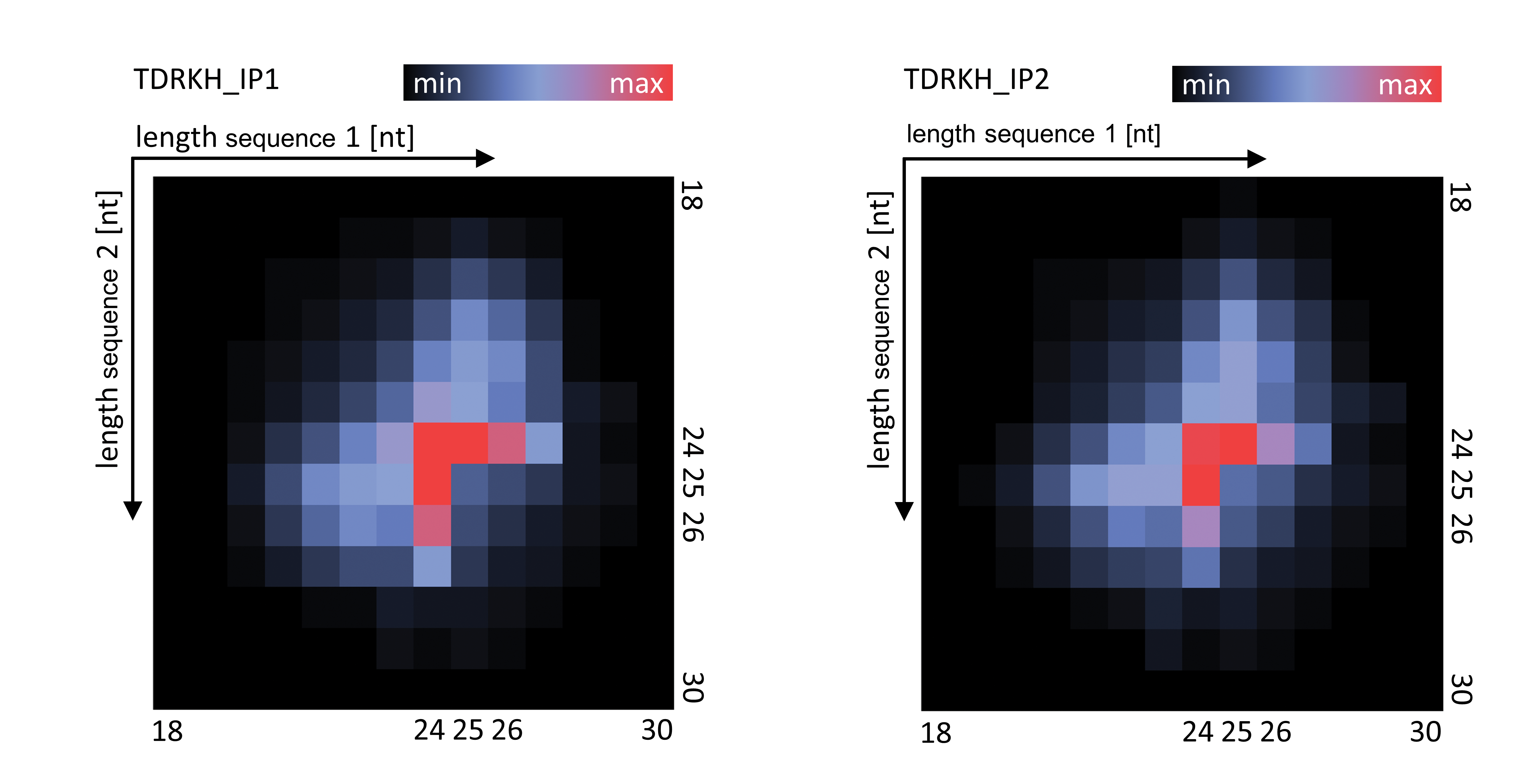

Supplement: Supplementary file 1 [file cells-09-01356-s001.zip › fig. S3.tif]

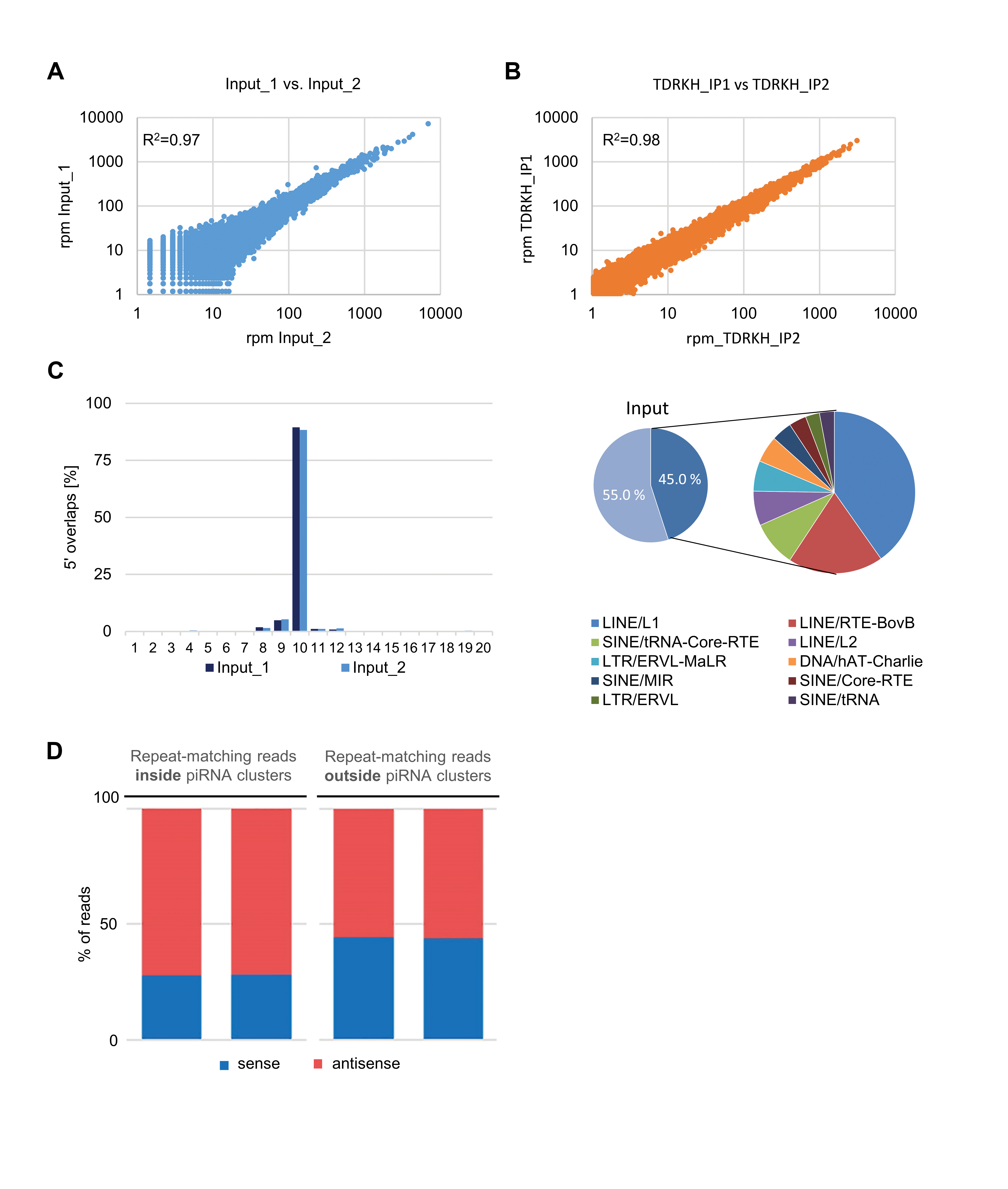

Supplement: Supplementary file 1 [file cells-09-01356-s001.zip › fig. S4.tif]

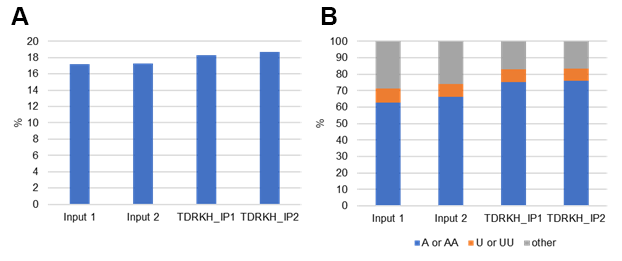

Supplement: Supplementary file 1 [file cells-09-01356-s001.zip › fig. S5.tif]
